# Ginsentides: Cysteine and Glycine-rich Peptides from the Ginseng Family with Unusual Disulfide Connectivity

**DOI:** 10.1038/s41598-018-33894-x

**Published:** 2018-11-01

**Authors:** James P. Tam, Giang K. T. Nguyen, Shining Loo, Shujing Wang, Daiwen Yang, Antony Kam

**Affiliations:** 1 School of Biological Sciences, Nanyang Technological University, 60 Nanyang Drive, 637551 Singapore, Singapore; 2 Department of Biological Sciences, National University of Singapore, 14 Science Drive 4, 117543 Singapore, Singapore; 3 Present Address: Wilmar International, 1, Research Link, Temasek Life Sciences Laboratory, Singapore, Singapore; 4 Present Address: School of Pharmaceutical Sciences & Collaborative Innovation Center for Diagnosis and Treatment of Infectious Diseases, Tsinghua University, 100084 Beijing, China

## Abstract

Ginseng, a popular and valuable traditional medicine, has been used for centuries to maintain health and treat disease. Here we report the discovery and characterization of ginsentides, a novel family of cysteine and glycine-rich peptides derived from the three most widely-used ginseng species: *Panax ginseng*, *Panax quinquefolius*, and *Panax notoginseng*. Using proteomic and transcriptomic methods, we identified 14 ginsentides, TP1-TP14 which consist of 31–33 amino acids and whose expression profiles are species- and tissues-dependent. Ginsentides have an eight-cysteine motif typical of the eight-cysteine-hevein-like peptides (8C-HLP) commonly found in medicinal herbs, but lack a chitin-binding domain. Transcriptomic analysis showed that the three-domain biosynthetic precursors of ginsentides differ from known 8C-HLP precursors in architecture and the absence of a C-terminal protein-cargo domain. A database search revealed an additional 50 ginsentide-like precursors from both gymnosperms and angiosperms. Disulfide mapping and structure determination of the ginsentide TP1 revealed a novel disulfide connectivity that differs from the 8C-HLPs. The structure of ginsentide TP1 is highly compact, with the N- and C-termini topologically fixed by disulfide bonds to form a pseudocyclic structure that confers resistance to heat, proteolysis, and acid and serum-mediated degradation. Together, our results expand the chemical space of natural products found in ginseng and highlight the occurrence, distribution, disulfide connectivity, and precursor architectures of cysteine- and glycine-rich ginsentides as a class of novel non-chitin-binding, non-cargo-carrying 8C-HLPs.

## Introduction

Of all medicinal herbs, ginseng is the most widely used and the most economically valuable. According to Baeg & Seung, in 2013 worldwide sales of ginseng and ginseng-derived products surpassed 200 million dollars^1^. A literature survey of PubMed and Google Scholar showed that ginseng is one of the most studied medicinal herbs with >10,000 scientific reports related to different aspects of ginseng research, including the medicinal benefits, phytochemistry, and cultivation of ginseng.

Ginseng is the collective name for 13 species of the *Panax* genus of the Araliaceae family^2^. Most studies involve three common and commercial-important ginseng species: *Panax ginseng* (Asian ginseng), *Panax quinquefolius* (American ginseng), and *Panax notoginseng* (notoginseng). The name *Panax* means “cure-all”, and ginseng has indeed been exploited for many uses, ranging from health maintenance to the treatment of diseases. In traditional Chinese medicine, ginseng is used to vitalize visceral organs, stimulate rapid recovery from illnesses, and improve blood circulation^3^. Ginseng is also used as an adaptogenic herb to maintain general well-being and counteract the effects of physical and emotional stress by enhancing memory, relieving fatigue, and improving stamina^4^.

Studies of active components in ginseng predominantly focused on small-molecule metabolites. To date, >200 chemical compounds have been identified from the three most common ginseng species^3,5^. The best known are ginsenosides that belong to the saponin family^3,5,6^. Ginsenosides can be broadly divided into two groups, protopanaxadiol and protopanaxatriol^6^. However, no multiple disulfide-constrained peptides with MW 2 to 6 kDa has been reported for ginseng species, even though they are commonly found in plant and play important roles in host defense as antimicrobials, insecticidals^7–11^, and proteinase inhibitors^12–15^. The primary amino acid sequences of plant-derived multiple disulfide-crosslinked peptides, also known as cysteine-rich peptides (CRPs), generally contain >16% cysteine residues that form between three and five disulfide bonds^16^. These multiple disulfide linkages confer resistance to degradation by heat, acid and enzymes^12,13,15,17^.

CRPs are classified into different families based on their cysteine motifs^16^. Our recent studies showed that many medicinal herbs contain a common cysteine motif with a tandemly-connecting cysteine in the third and fourth position. An example is the eight-cysteine hevein-like peptides (8C-HLPs) that have a cysteine motif arranged as CX_n_CX_n_CCX_n_CX_n_CX_n_CX_n_C^16,18–20^. The prototypic member of 8C-HLPs is hevein, which was first isolated from the rubber tree (*Hevea brasiliensis*) and contains cystine-knot disulfide connectivity as well as a chitin-binding domain that promotes binding to chitin which is found in fungi and insects^21^.

Here we report the identification, isolation, and characterization of a novel family of 8C-HLPs that lack a chitin-binding domain, termed ginsentides. We identified ginsentides TP1-TP14 from three common ginseng species, *Panax ginseng*, *Panax quinquefolius*, and *Panax notoginseng* of the Araliaceae family. Proteomic analysis showed that the ginsentides contain the cysteine motif present in the 8C-HLP family, but disulfide mapping, NMR structural determination and transcriptomic analysis showed that these peptides display an unusual disulfide connectivity and precursor architecture. Additionally, the ginsentides have a high cysteine and glycine content that accounts for >50% of the amino acids present in their sequences. The high cysteine content (>24%) together with cystine residues at both the N- and C-termini of ginsentides confers a tightly folded pseudocyclic structure. We also showed that ginsentides are stable against heat, acidic, proteolytic and human-serum-mediated degradation. Taken together, our discovery of ginsentides unveiled a novel family of underexplored cysteine-rich peptides derived from ginseng that could have therapeutic importance.

## Results

### Identification of cysteine-rich peptides from three ginseng species: Panax ginseng, Panax quinquefolius and Panax notoginseng

**Figure 1:**
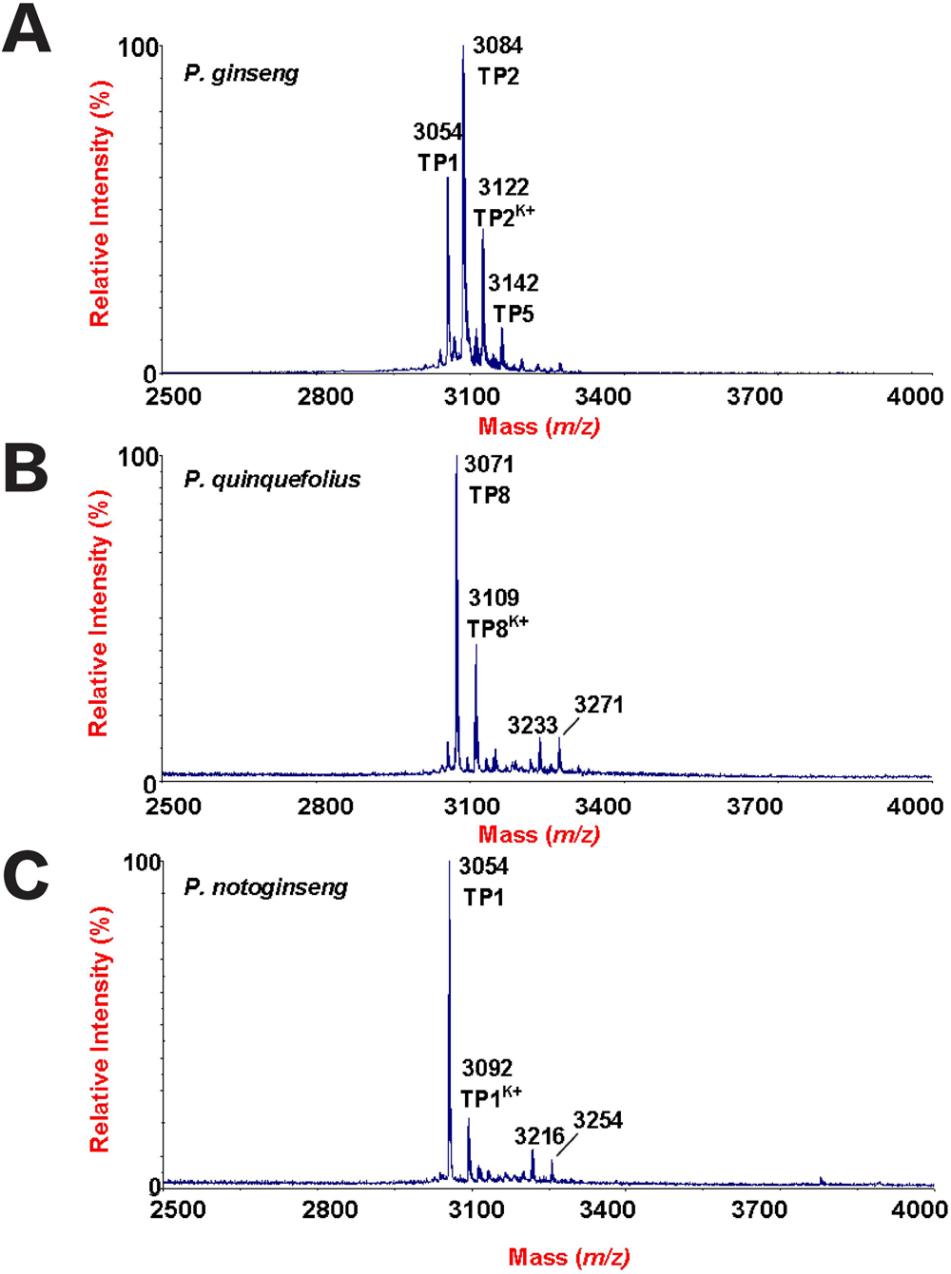
Mass spectrometry profiles of aqueous extracts of roots from (**A**) *Panax ginseng*, (**B**) *Panax quinquefolius* and (**C**) *Panax notoginseng* using MALDI-TOF MS.

**Figure 2:**
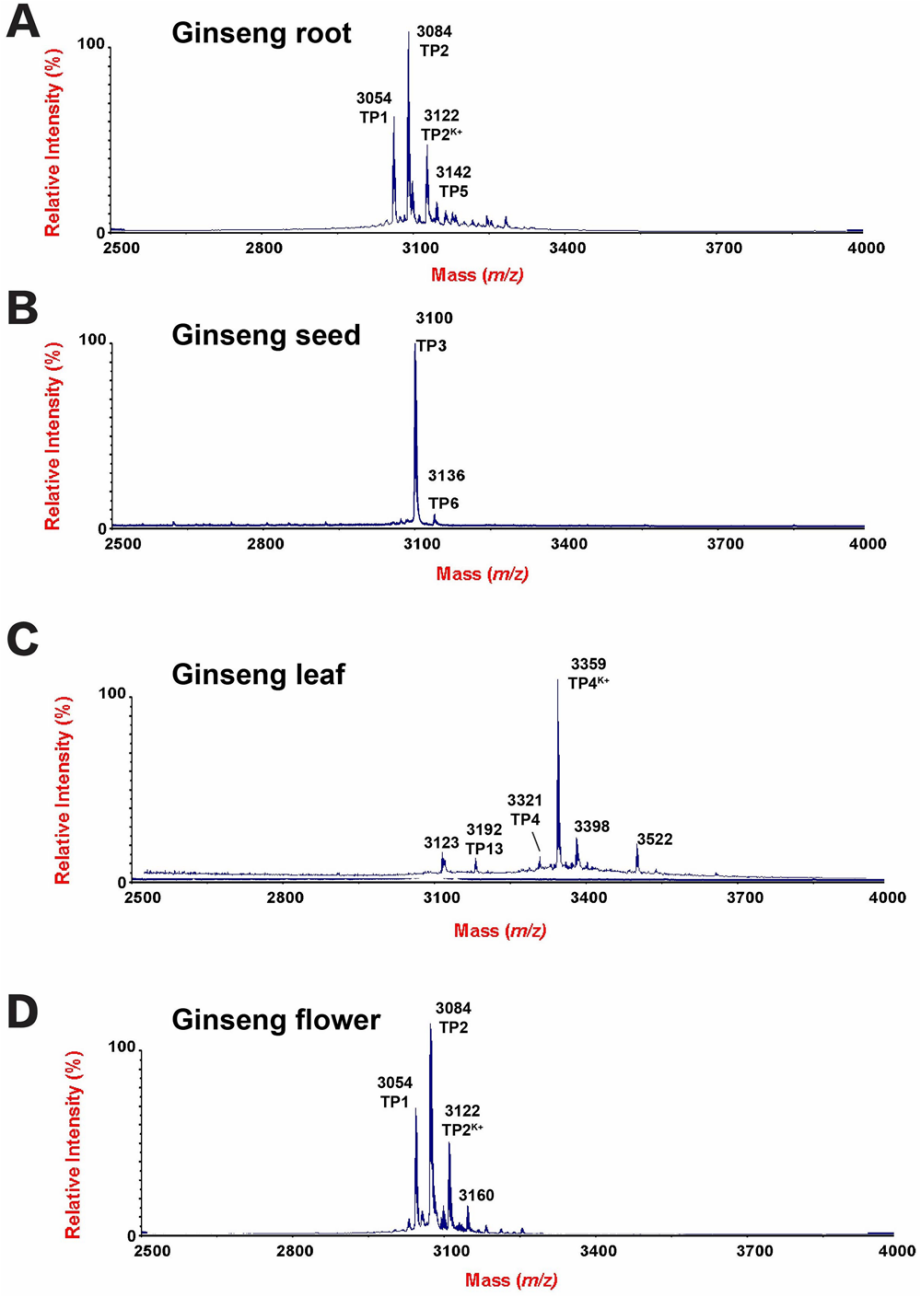
Mass spectrometry profiles of aqueous extracts of (**A**) roots, (**B**) seeds, (**C**) leaves and (**D**) flowers from *Panax ginseng* using MALDI-TOF MS.

**Figure 3:**
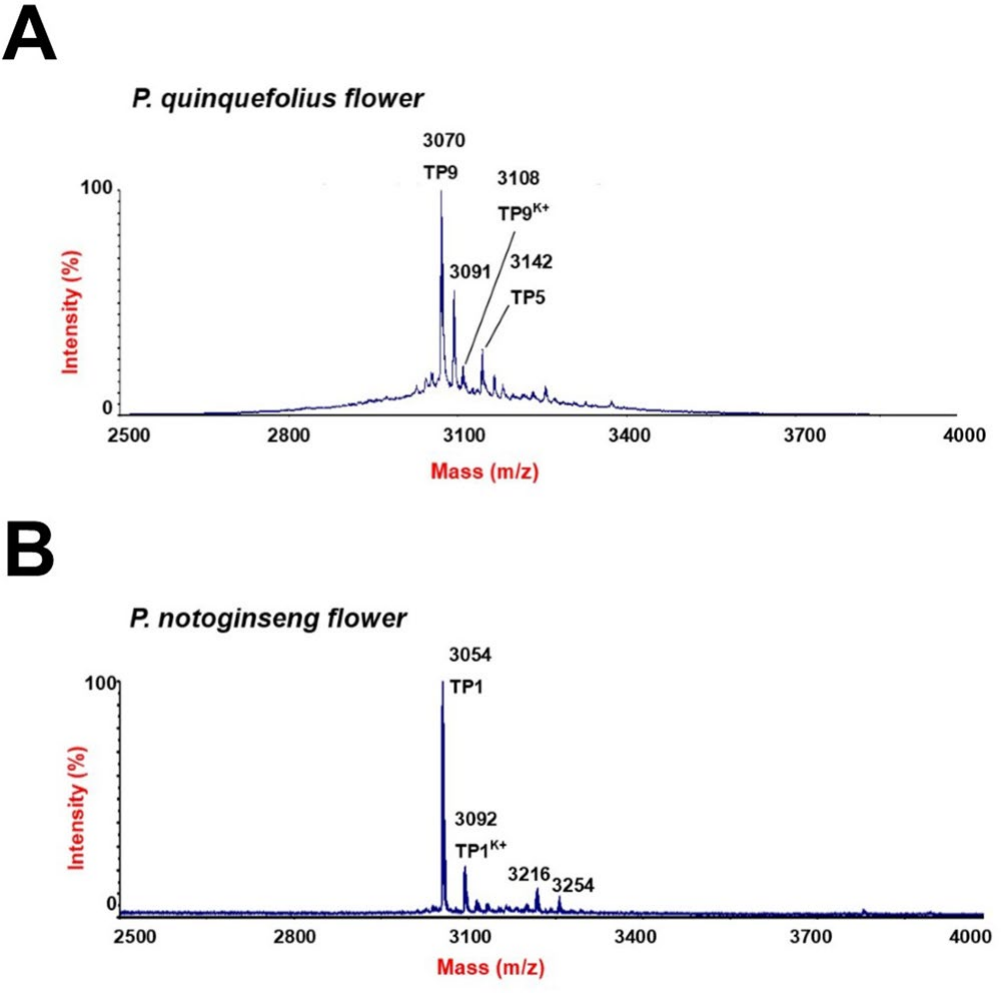
Mass spectrometry profiles of aqueous extracts of (**A**) *Panax quinquefolius* and (**B**) *Panax notoginseng* flowers using MALDI-TOF MS.

A mass spectrometry-driven profiling of aqueous extracts of *Panax ginseng*, *Panax quinquefolius*, and *Panax notoginseng* roots revealed a cluster of strong signals in the mass range of 3–5 kDa (Fig. 1). Mass spectra of the extracts showed that each species expressed a unique set of mass signals with *m/z* of 3000–3500. *Panax ginseng* displayed four strong peaks having *m/z* values of 3054, 3084, 3122, and 3142, as compared to 3054, 3092, 3216 and 3254 for *Panax notoginseng*, and 3071, 3109, 3233, and 3271 for *Panax quinquefolius* (Fig. 1). Figure 2 shows the tissue distribution of ginsentides in *Panax ginseng* roots, seeds, leaves and flowers. Figure 3 shows the mass spectra of *Panax notoginseng* and *Panax quinquefolius* flower. The peak at *m/z* value 3054 was designated as ginsentide TP1, which was isolated by RP-HPLC and subjected to *S-*reduction and *S-*alkylation using dithiothrietol (DTT) and N-ethylmaleimide (NEM) to determine the disulfide content. All other peaks representing the putative ginsentides in the mass region 3 to 3.5 kDa showed a mass shift of 1008 Da after *S-*reduction and *S-*alkylation, indicating the presence of eight cysteine residues (Supplementary Data S1).

### Primary sequence and biosynthesis of ginsentides

**Figure 4:**
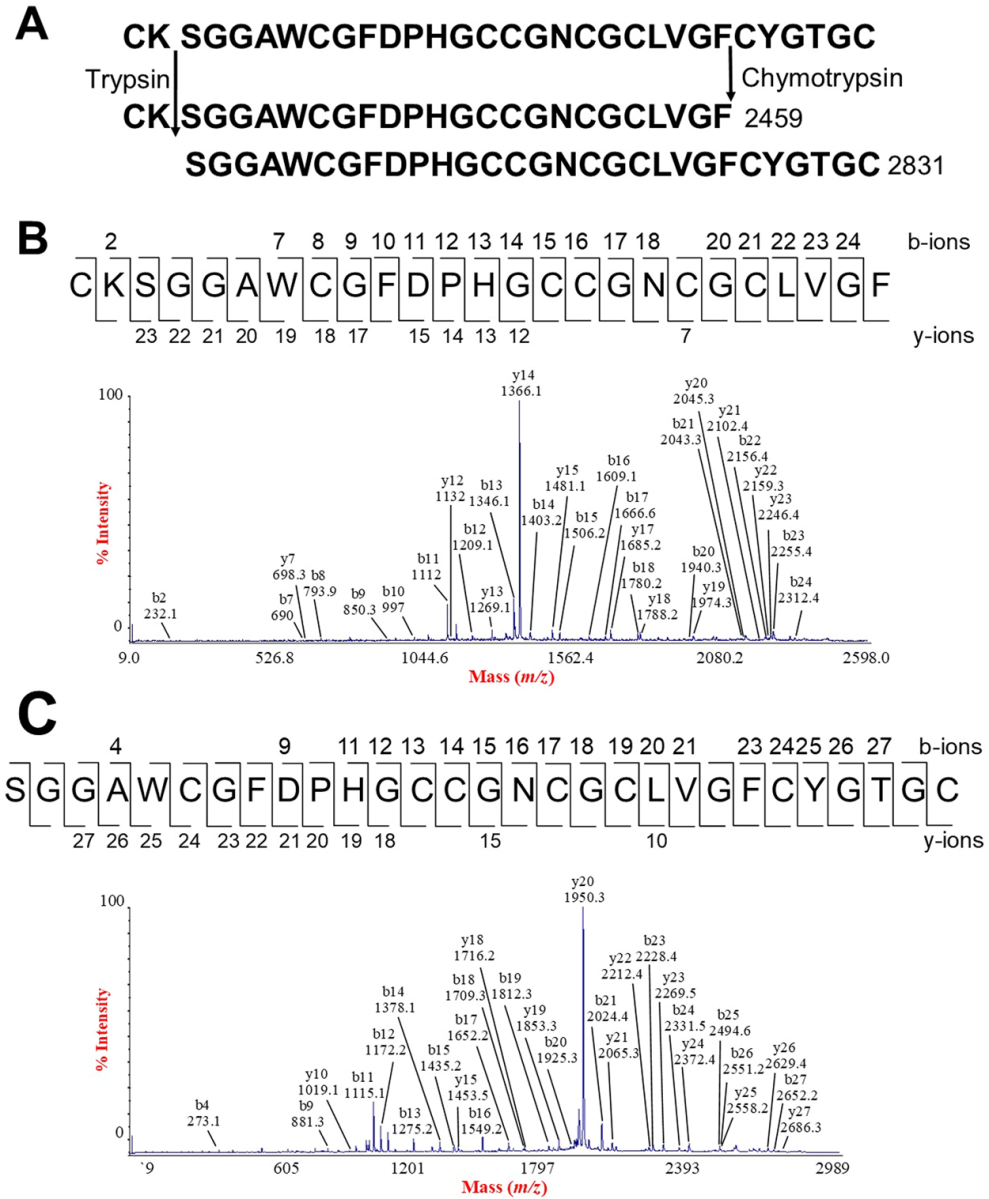
*De novo* sequencing of ginsentide TP1. Enzymatic digestion of *S*-reduced peptides by chymotrypsin and trypsin generated one major fragment each with m/z values of 2459 and 2831, respectively. The sequences of the fragments were deduced using the *b*-ions and *y*-ions generated from MALDI-TOF MS/MS.

MS/MS sequencing of the 3054 Da ginsentide TP1, which was found in both *Panax ginseng* and *Panax notoginseng*, serves as a representative example of the TP peptides (Fig. 4). Enzymatic digestion of *S*-reduced TP1 by chymotrypsin or trypsin produced one major fragment having *m/z* values of 2459 and 2831, respectively (Fig. 4). Using the *b*-ions and *y*-ions generated from MALDI-TOF MS/MS, these fragments showed that the sequence of the 2459-fragment was CKSGGAWCGFDPHGCCGNCGCLVGF and the 2831-fragment was SGGAWCGFDPHGCCGNCGCLVGFCYGTGC. Combining these two overlapping fragments yielded the full sequence of the 3054-Da ginsentide TP1. *De novo* peptide sequencing was also performed to determine the primary sequence of the 3084-Da ginsentide TP2 (Supplementary Data S2).

**Figure 5:**
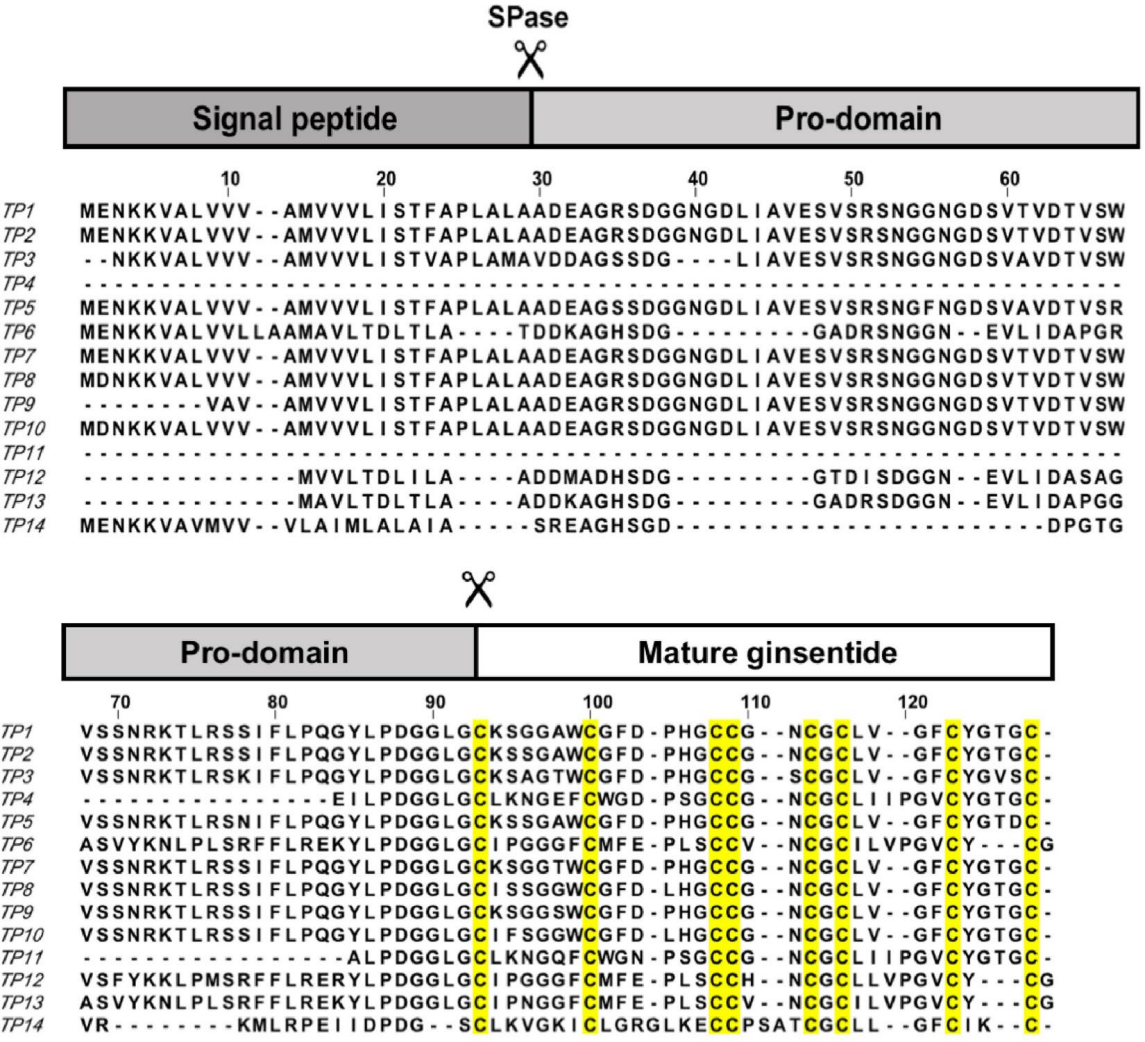
Ginsentide-encoding transcripts from *Panax ginseng*, *Panax quinquefolius* and *Panax notoginseng* deduced from *de novo* assembly of transcriptome data from the NCBI database. The transcriptome data used are listed as follows: *Panax notoginseng flower* (SRX378878), *Panax quinquefolius flower* (SRX062267), *Panax ginseng flower* (SRX181263), *Panax ginseng flower* (SRX378873), *Panax notoginseng leaf* (SRX378880), *Panax quinquefolius seed* (SRX529365), *Panax ginseng root* (ERX137460). SPase: signal peptidase.

The Basic Local Alignment Search Tool (BLAST) of the NCBI database and transcriptomic analyses revealed that ginsentides are the mature products of ginseng-specific abundant proteins (GSAPs). Our results revealed 14 putative ginsentide-encoding gene sequences (TP1-TP14) from the *Panax* family of *Panax ginseng*, *Panax quinquefolius and Panax notoginseng* (Fig. 5 and Table 1). Sequence analysis also showed that the eight cysteine residues in the C-terminal regions of ginsentide-encoding genes are conserved. All ginsentides have between 31 and 33 amino acids that include eight cysteine residues arranged in a cysteine motif of CX_n_CX_n_CCX_n_CXCX_n_CX_n_C with the tandemly connecting CC motif highlighted in bold. In addition, ginsentides are exceptionally glycine-rich; TP1 has nine glycine residues. Sequence comparison showed that 66% of amino acid residues in TP1 are conserved among the TP family, and sequence conservation is highest for cysteine and glycine. Transcriptomic analysis further showed that ginsentides (TP1-TP14) are synthesized as precursors with three domains: N-terminal signal peptide, pro-domain and C-terminal mature ginsentides (Fig. 5).

**Table 1:** Sequence alignment of ginsentide TP1-TP14.

Ginsentide	Amino acid sequence	Calculated Mass (m/z)	mRNA*
TP1	CKSGGAWCGFD-PHGCCG--NCGCLV--GFCYGTGC-	3053.10	PG, PN
TP2	CKSSGAWCGFD-PHGCCG--NCGCLV--GFCYGTGC-	3083.11	PG, PQ
TP3	CKSAGTWCGFD-PHGCCG--SCGCLV--GFCYGVSC-	3098.15	PG, PQ
TP4	CLKNGEFCWGD-PSGCCG--NCGCLIIPGVCYGTGC-	3320.31	PG
TP5	CKSSGAWCGFD-PHGCCG--NCGCLV--GFCYGTDC-	3141.12	PG, PQ
TP6	CIPGGGFCMFE-PLSCCV--NCGCILVPGVCY--CG	3135.27	PG, PQ
TP7	CKSGGTWCGFD-PHGCCG--NCGCLV--GFCYGTGC-	3083.11	PG
TP8	CISSGGWCGFD-LHGCCG--NCGCLV--GFCYGTGC-	3070.12	PQ
TP9	CKSGGSWCGFD-PHGCCG--NCGCLV--GFCYGTGC-	3069.10	PQ
TP10	CIFSGGWCGFD-LHGCCG--NCGCLV--GFCYGTGC-	3130.15	PQ
TP11	CLKNGQFCWGN-PSGCCG--NCGCLIIPGVCYGTGC-	3318.34	PQ
TP12	CIPGGGFCMFE-PLSCCH--NCGCLLVPGVCY--CG	3173.26	PQ
TP13	CIPNGGFCMFE-PLSCCV--NCGCILVPGVCY--CG	3192.29	PN
TP14	CLKVGKICLGRGLKECCPSATCGCLL--GFCIK--C-	3310.61	PQ

*PG: *Panax ginseng*; PN: *Panax notoginseng*; PQ: *Panax quinquefolius*.

### Secondary structure and disulfide connectivity of ginsentide TP1

**Figure 6:**
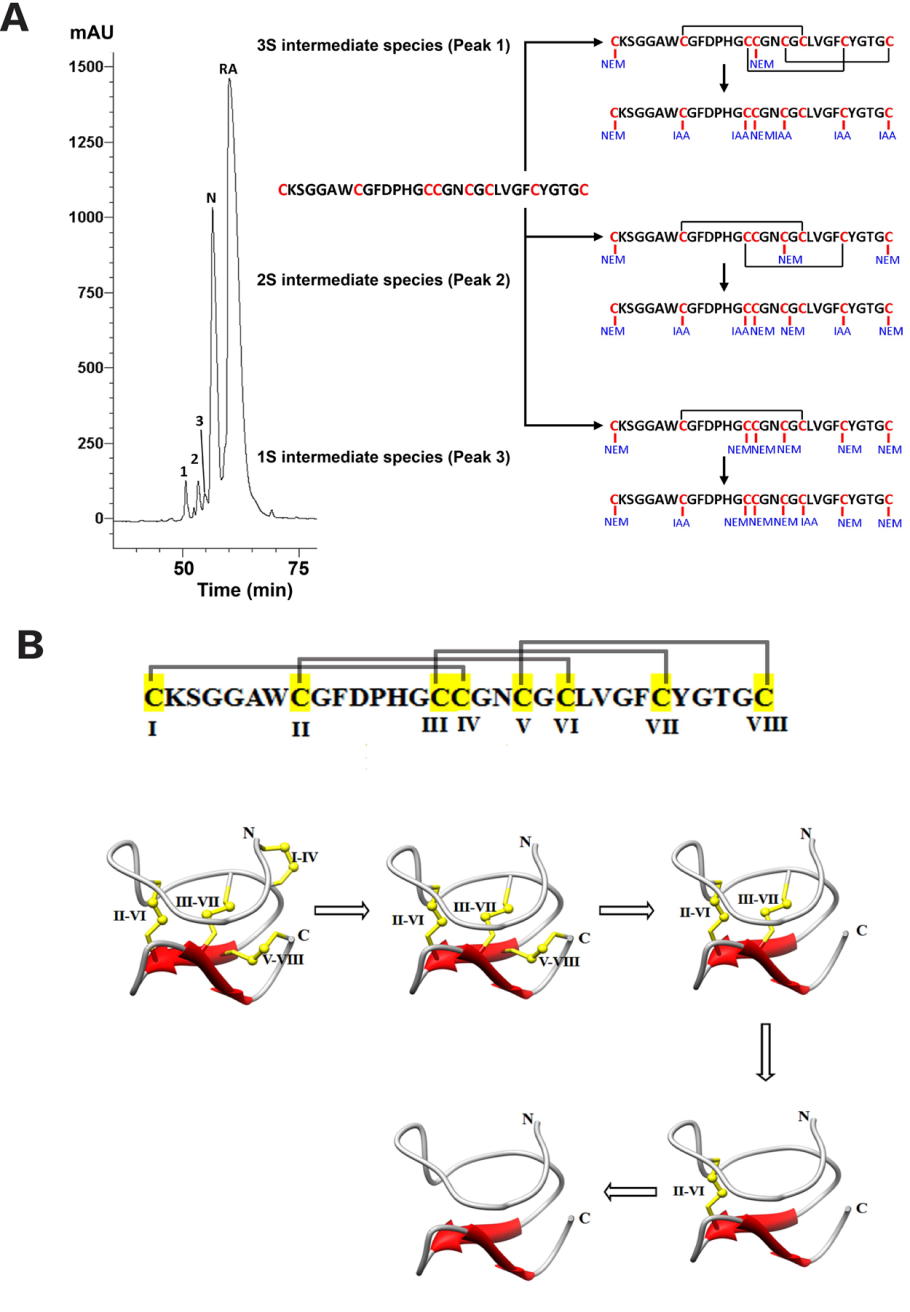
(**A**) HPLC profile of partially *S*-reduced and *S*-alkylated ginsentide TP1. Peaks 1, 2, 3, N, and RA contained the 3*SS*, 2*SS*, 1*SS*, native peptide, and fully *S*-NEM alkylated peptides, respectively. A schematic representation of ginsentide TP1 disulfide mapping is also shown; (**B**) The putative unfolding pathway of ginsentide TP1 as determined by disulfide connectivity mapping. Under our experimental conditions, the Cys I-IV bond was the first to be reduced to generate the 3*SS* species, followed by the Cys V-VIII bond, generating the 2*SS* species, then the Cys III-VII bond generating the 1*SS* species, and lastly, the Cys II-VI bond.

We next used a chemical mapping method involving sequential *S*-tagging to determine disulfide connectivity of ginsentides^22–26^. Stepwise determination of ginsentide TP1 to determine disulfide connectivity showed an initial partial *S*-reduction with tris(2-carboxyethyl)phosphine followed by *S*-alkylation with excess NEM (Fig. 6). Three NEM-labeled intermediates with one (1SS), two (2SS), or three (3SS) intact disulfide bonds were then collected. These intermediate species were subsequently fully *S*-reduced and *S*-tagged with a second alkylation reagent, iodoacetamide (IAM). Mixed *S*-labeled peptides were digested with trypsin and sequenced by MS/MS (Supplementary Data S3). Combining the information from the 1SS- and 3SS-intermediates, we deduced the ginsentide TP1 disulfide connectivity as Cys I-IV, Cys II-VI, Cys III-VII and Cys V-VIII.

### Tertiary structure of ginsentide TP1

**Figure 7:**
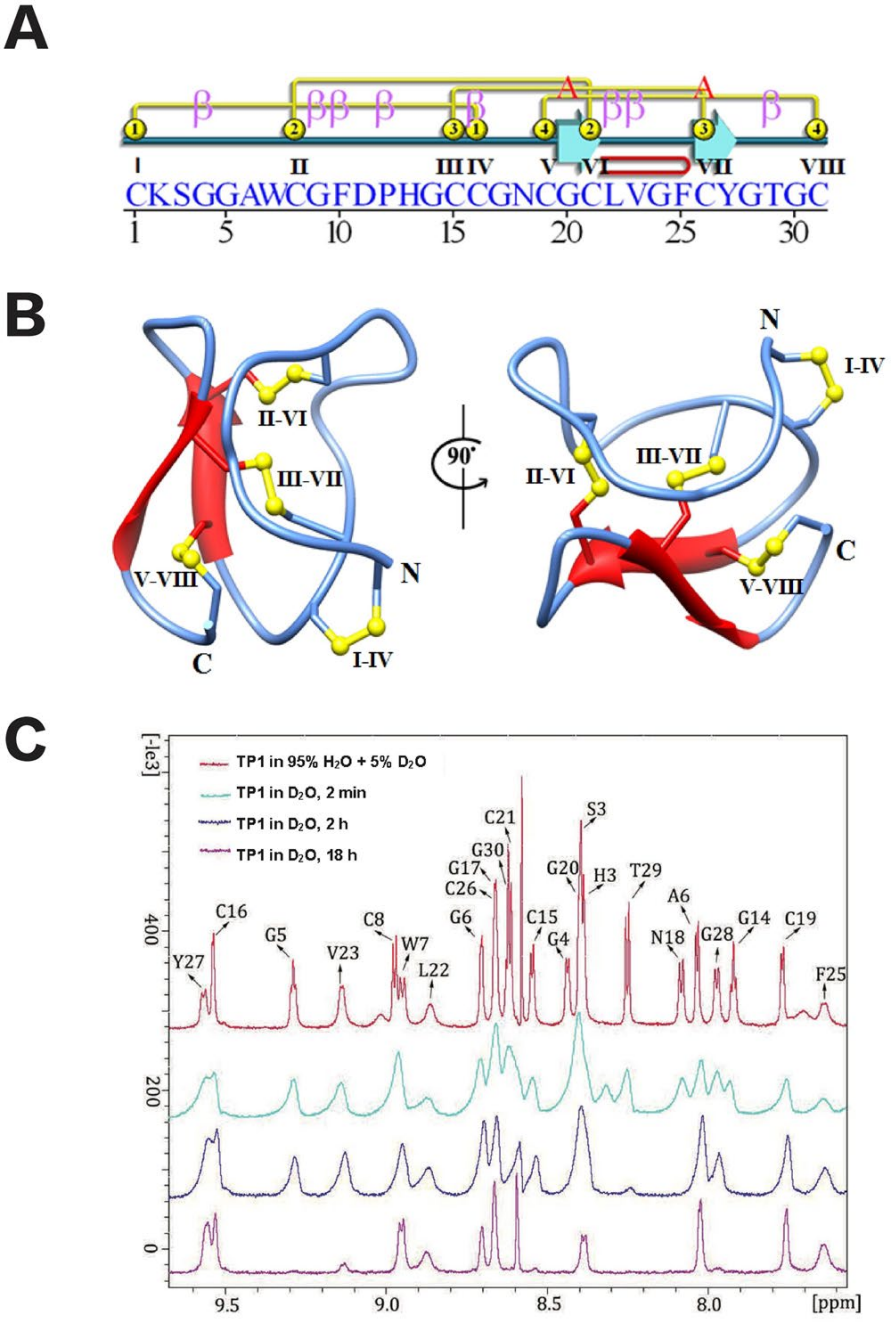
Ginsentide TP1 NMR structure. (**A**) Illustration of the structure topology against the ginsentide TP1 sequence. (**B**) Cartoon view of the ginsentide TP1 solution structure, with disulfide bonds colored yellow. (**C**) Comparison of the ^1^D spectra of ginsentide TP1 in 95% H_2_O/5% D_2_O and 100% D_2_O in the range of 7.6–9.6 ppm. Peaks of amide protons are labeled with assignments, except those from side chains. Red line represents TP1 in 95% H_2_O/5% D_2_O, and cyan, blue and magenta lines illustrate TP1 in 100% D_2_O for 2 min, 2 hr and 18 hr, respectively, at 25 °C.

The three-dimensional (3-D) structure of ginsentide TP1 was determined using the distance, dihedral angle and hydrogen bond restraints derived from ^1^H NMR analysis (Table 2). The average RMSD for secondary structural regions were 0.35 ± 0.05 Å and 0.68 ± 0.07 Å for all backbone and heavy atoms, respectively. Ginsentide TP1 (PDB code: 2ML7) adopts a β-sheet structure with two antiparallel β-strands consisting of residues Gly20-Leu22 and Phe25-Tyr27, and eight β-turns, as well as a β-hairpin that includes Gly20 to Tyr27 (Fig. 7A). The solution structures of ginsentide TP1 showed that it adopts unusual disulfide connectivity wherein the three disulfide bonds Cys I-IV, II-VI and III-VII adopt a cystine-knot fold similar to knottin family peptides such as the cystine-knot α-amylase inhibitors (Fig. 7B)^12,13,15,17,20^. The additional disulfide bond at Cys V-VIII is a penetrating disulfide bond that is unique to ginsentides. The overall structure is tightly folded with approximately 90% and 30% of the amide proton signals remaining in the ^1^D spectra after H/D exchange in D_2_O for 2 h and 18 h, respectively (Fig. 7C).

**Table 2:** NMR experimental and structural statistics of ginsentide TP1.

NOE constraints	551
Intra-residue (|i-j| = 0)	32
Sequential (|i-j| = 1)	220
Medium-range (1 < |i-j| < 5)	73
Long-range (|i-j|$\ge$ 5)	226
Dihedral angle restraints	13
Hydrogen bonds	9
PROCHECK-NMR Ramachandran plot (%)	
Most favored region	52.6
Additionally allowed region	47.4
Generously allowed region	0
Disallowed region	0
Average maximum violations per structure	
Distance (Å)	0.015 ± 0.002
van der Waals (Å)	2.2 ± 0.4
Torsion angles (°)	1.25 ± 0.12
CYANA target function value (Å^2^)	0.68 ± 0.13
Average RMSD to mean structure (Å)	
All back bone atoms (1--31)	0.35 ± 0.05
All heavy atoms (1--31)	0.68 ± 0.07

**Figure 8:**
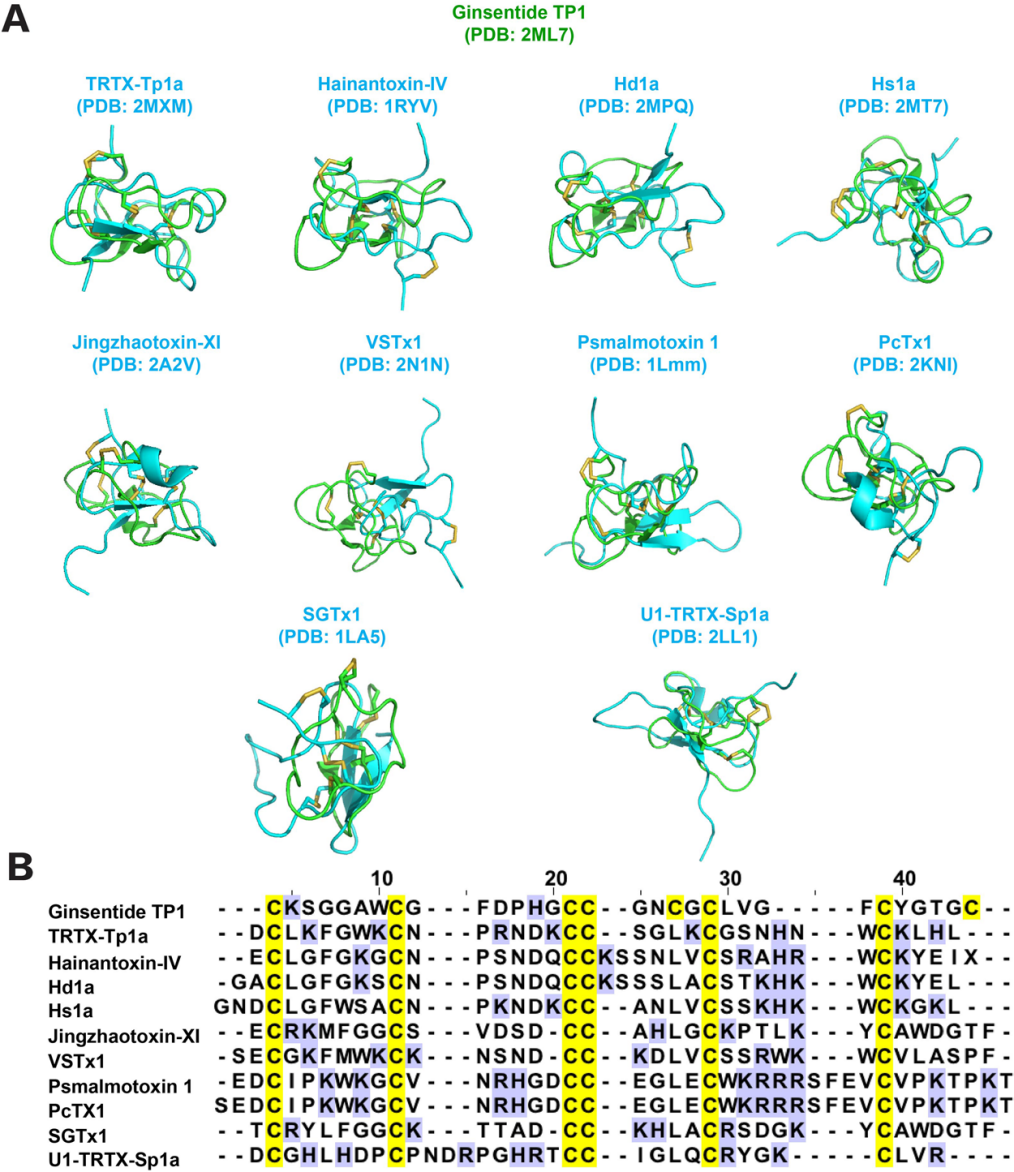
(**A**) Structure and (**B**) sequence alignment of ginsentide TP1 (PDB code: 2ML7) with spider toxins: hainantoxin-IV (PDB code: 1niy and 1ryv), Hs1A (PDB code: 2mt7), U1-TRTX-Sp1a (PDB code: 2LL1), jingzhaotoxin-XI (PDB code: 2a2v), TRTX-Tp1a (PDB code: 2mxm), Hd1a (PDB code:2mpq), psalmotoxin-1 (PDB code: 2kni), PcTX1 (PDB code:1lmm), SGTx1 (PDB code:1la4), and VSTx1 (PDB code:2n1n). Cysteine residues are highlighted in yellow whereas charged residues are highlighted in blue.

NMR analysis showed that ginsentides possess a pseudocyclic structure in which both N- and C-terminal Cys residues participate in the disulfide linkages. This arrangement, together with a cystine-knot, forms the ginsentide sulfur core. A search for conserved structures using the ginsentide TP1 coordinates in the Dali Server^27^ yielded 11 similar structures with Z-scores ranging from 2.0 to 2.6. All 11 structures belong to ion (sodium/potassium/calcium) channel blockers from spider toxins, such as hainantoxin-IV (PDB code: 1niy and 1ryv)^28^, HS1A (PDB code: 2mt7), U1-TRTX-SP1A (PDB code: 2LL1)^29^, jingzhaotoxin-XI (PDB code: 2a2v)^30^, μ-TRTX-Tp1a (PDB code: 2mxm)^31^, HD1A (PDB code:2mpq)^32^, psalmotoxin-1 (PDB code: 2kni)^33^, psalmotoxin 1 (PDB code:1lmm)^34^, SGTX1 (PDB code:1la4)^35^, and VSTx1 (PDB code:2n1n)^36^ (Fig. 8A). The primary sequence similarities of ginsentide TP1 and the 17 spider toxins are limited to the six cysteine residues involved in the disulfide bonds: Cys I-IV, Cys II-VI, and Cys III-VII (Fig. 8B). These three disulfide bonds form a scaffold that is similar to the common cystine-knot disulfide connectivity^12,13,15^. Ginsentide TP1 is unique in the presence of an additional disulfide bond that links the C-terminal Cys VIII to Cys V in the middle of the peptide sequence. Analyses of the peptide surface properties revealed the presence of positively charged residues distributed around the hydrophobic patches on the structural surface of spider toxins that are essential for their ion-channel blocking properties^28,33,37–39^. This positively-charged surface property, however, is absent in ginsentide TP1, where more than half of its sequence are Cys and Gly residues.

### Stability of ginsentide TP1 against heat, proteolytic, acid and serum-mediated degradation

**Figure 9:**
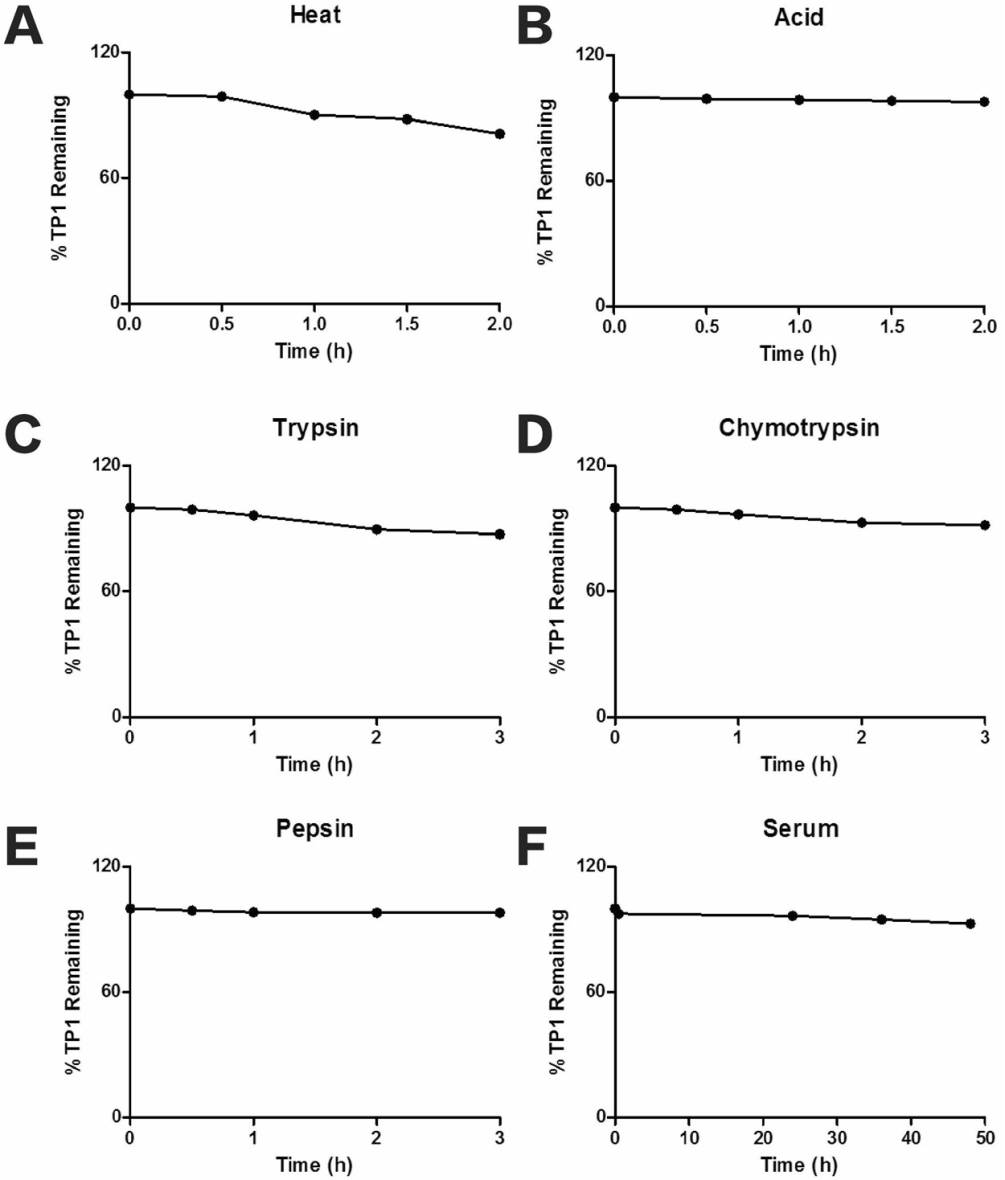
(**A**) Heat, (**B**) acid, (**C**) trypsin, (**D**) chymotrypsin, (**E**) pepsin and (**F**) serum stability of ginsentide TP1. All results are expressed as mean ± S.E.M. (n = 3).

To examine the stability of the unique pseudocyclic cystine-knot motif of ginsentides, heat, proteolytic, acid and serum stability assays were performed on ginsentide TP1 (Fig. 9). The percentages of remaining ginsentides were quantified based on their relative peak areas in RP-HPLC profiles before and after treatment. Ginsentide TP1 was relatively stable to heat with less than 10% degradation after heating at 100 °C for 30 min and 29% after 120 min. Ginsentide TP1 displayed high stability against enzymatic degradation, including that by trypsin, chymotrypsin, and pepsin, with >80% of peptides remaining intact after 3 h incubation. Similarly, in an acid stability assay, ginsentide TP1 was highly stable in 0.2 N HCl. Ginsentide TP1 was also stable in human serum with <10% degradation over a 48 h incubation period at 37 °C.

### Cytotoxicity, hemolyticity and immunogenicity assessment of ginsentides TP1

**Figure 10:**
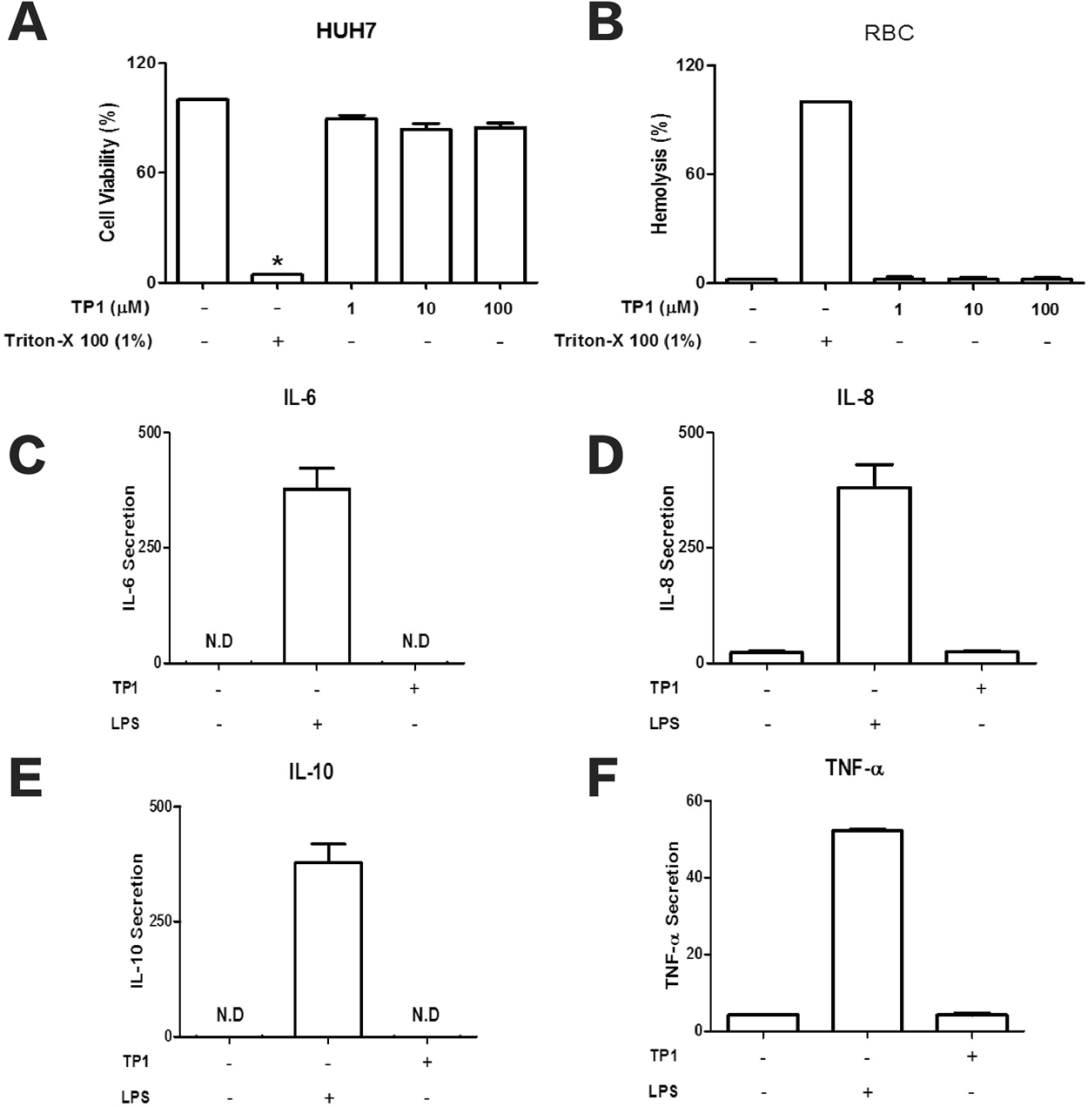
Ginsentide TP1 shows no (**A**) cytotoxic activities in Huh7 cells or (**B**) hemolytic effects. Ginsentide TP1 does not induce (**C**) IL-6, (**D**) IL-8, (**E**) IL-10, and (**F**) TNF-α release from THP-1 cells. LPS was used as a positive control. All results are expressed as mean ± S.E.M. (n = 3). *P < 0.05 compared to control group.

To examine the toxicity of ginsentides, we incubated ginsentide TP1 with Huh7 cells or red blood cells and found no change in cell viability or hemolysis at concentrations up to 100 µM. Ginsentide TP1 was non-immunogenic to THP-1 cells and induced no observable increase in IL-6, IL-8, IL-10 and TNF-α secretion (Fig. 10).

### Transcriptomic database search for ginsentide-like 8C-HLPs

**Figure 11:**
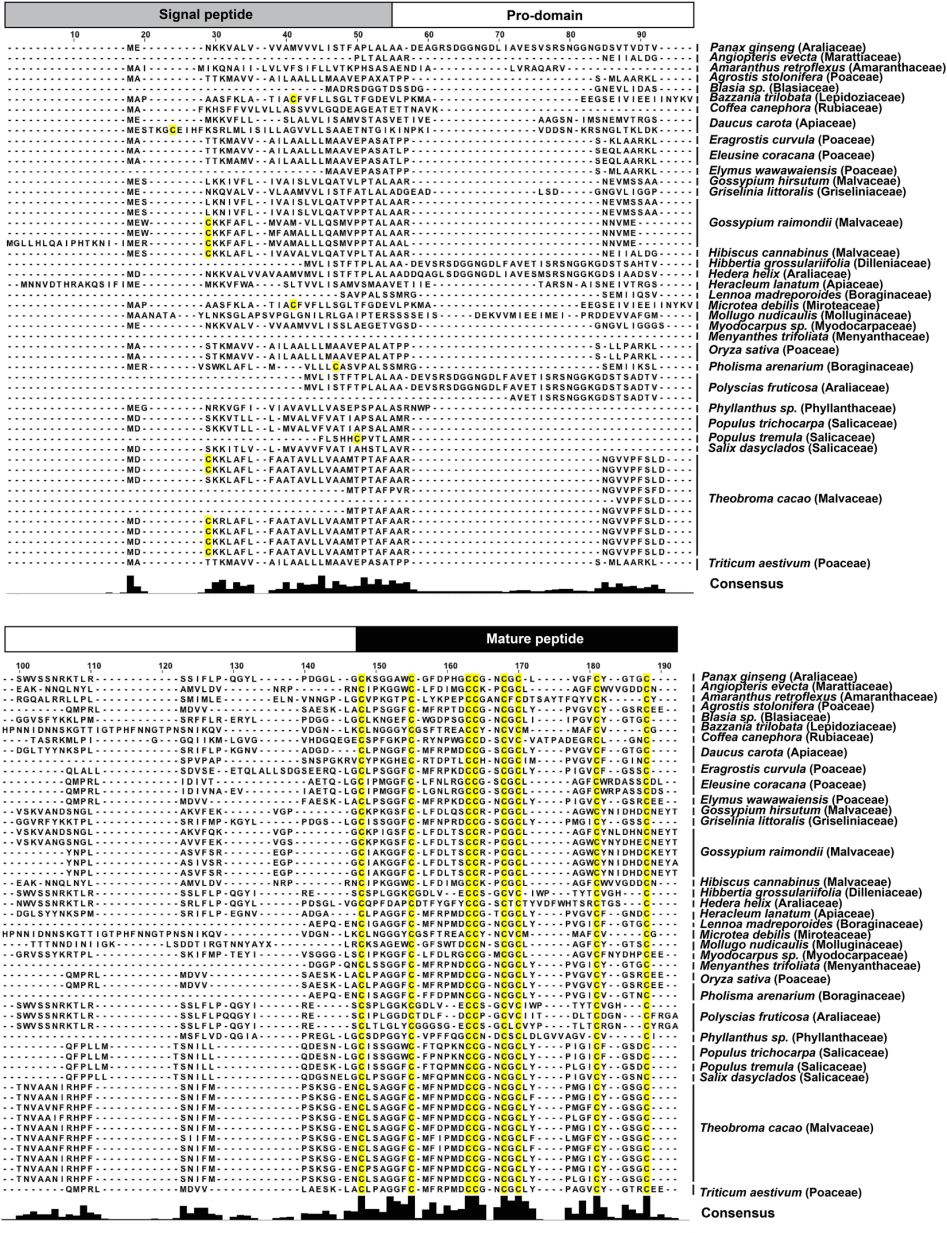
Alignment of 51 three-domain precursor sequences with four disulfide bonds and a cysteine motif of CXnCXnCCXnCXCnCXnC by TBLASTN and BLASTP search of the Onekp and NCBI databases.

To explore the occurrence and distribution of ginsentide-like 8C-HLPs in other plant species, we performed a TBLASTN and BLASTP search of the NCBI and Onekp databases using the ginsentide TP1 precursor sequence. Based on our database search, we identified 50 other three-domain ginsentide-like precursor sequences containing four disulfide bonds and a cysteine motif of CX_n_CX_n_CCX_n_CXCX_n_CX_n_C from 31 plant species in 19 families (Fig. 11).

## Discussion

In this study, we report the identification, isolation and characterization of 14 novel ginseng-derived cysteine-rich peptides, ginsentides TP1-TP14, from *Panax ginseng*, *Panax quinquefolius*, and *Panax notoginseng*. To the best of our knowledge, this is the first report on the discovery and characterization of ginseng-derived CRPs.

Using transcriptomic and proteomic approaches, we collectively identified 14 ginsentides (TP1-TP14). Ginsentides are 3 to 3.5 kDa peptides with 31–33 amino acids that are rich in Cys and Gly residues. With cysteine occurring at approximately one in every four amino acids, ginsentides are highly disulfide constrained and structurally compact. All ginsentides possess a CX_6_CX_6–7_CCX_2–4_CXCX_4–6_CX_1–4_C cysteine motif that is similar to 8C-HLPs. However, the 8C-cysteine motif of ginsentides differs from other 8C-HLPs in that it contains both a CC and a CXC motif. This cysteine motif results in a fold that contains one loop with a single amino acid and five loops of >2 amino acids. Additionally, all 14 ginsentides had high sequence similarity with conservation of cysteine and glycine residues. In particular, the intercysteine loop 4 is absolutely conserved in terms of loop size and presence of a Gly residue. In contrast, loop 5 and loop 6 showed a greater variability in size, particularly for ginsentides TP6, TP12, TP13 and TP14.

Interestingly, although ginsentide sequences have high sequence similarity (>66%), the occurrence and distribution patterns of ginsentides are species-dependent. At the mRNA level, ginsentide TP4 and TP7 are unique to *Panax ginseng*, whereas only *Panax quinquefolius* expresses ginsentides TP8, TP9, TP10, TP11, TP12 and TP14. Ginsentide TP13 is unique to *Panax notoginseng* and ginsentides TP2, TP3, TP5 and TP6 are common to both *Panax ginseng* and *Panax quinquefolius*. TP1 is produced by both *Panax ginseng* and *Panax notoginseng*. Mass spectrometry profile analyses revealed that ginsentide expression is also tissue-dependent. Aqueous extracts of roots and flowers from *Panax ginseng* displayed similar ginsentide expression patterns, with TP1 and TP2 as the dominant ginsentides. *Panax quinquefolius* and *Panax notoginseng* also had similar expression profiles in aqueous extracts of roots and flowers. In *Panax ginseng*, we saw a distinct ginsentide tissue expression pattern wherein the dominant ginsentide in aqueous extracts of seeds and leaves was TP3 and TP4, respectively. Collectively, these results suggested that ginsentide expression profiles could be used as biologic markers for identifying species and tissues of ginseng.

The 8C-HLPs belong to a family of CRPs that has an evolutionarily conserved CX_n_CX_n_CCX_n_CX_n_CX_n_CX_n_C cysteine motif. The tandemly-connecting CC motif at Cys III and Cys IV found in both 6C-HLPs and 8C-HLPs produce the cystine-knot disulfide connectivity of Cys I-IV, Cys II-V and Cys III-VI. For 8C-HLPs, the cysteine knot is followed by the small intercysteine loop Cys VII-VIII. The 8C-HLPs can be further divided into two subfamilies based on the presence or absence of a chitin-binding domain. Chitin-binding 8C-HLPs have a highly conserved SXΦXΦ domain (Φ, aromatic residues; X, any amino acid) in intercysteine loop 3 and a conserved aromatic residue at loop 4, which are essential for chitin-binding activity^18,19,21^. Because ginsentides lack the chitin-binding domain, we have classified them into a new subfamily described as non-chitin-binding 8C-HLPs. Transcriptome database searches of NCBI and Onekp revealed that 31 other plant species from 19 families in both gymnosperms and angiosperms express 50 other three-domain ginsentide-like 8C-HLP precursor sequences having the cysteine motif of CX_n_CX_n_CCX_n_CXCX_n_CX_n_C. Ginsentide-like peptides are found in some of our most important crops, including coffee (*Coffea canephora*), cacao (*Theobroma cacao*), cotton (*Gossypium raimondii*), rice (*Oryza sativa*) and wheat (*Triticum aestivum*).

Although ginsentides display a cysteine spacing pattern typical of 8C-HLPs with a tandemly connecting CC motif, ginsentides display a novel disulfide connectivity not found in 8C-HLPs. Using the stepwise *S*-reduction and *S*-alkylation method reported by Gray *et al*.^22^, we unequivocally determined the connectivity of ginsentide TP1 as Cys I-IV, II-VI, III-VII and V-VIII. The disulfide bonds Cys I-IV, II-VI, and III-VII formed a cystine-knot that is similar to that of 6C-HLPs, whereas the fourth penetrating disulfide bond Cys V-VIII is unique to ginsentides. By comparing differences in cysteine spacing patterns and disulfide connectivities between ginsentides and chitin-binding 8C-HLPs, we found that ginsentides and ginsentide-like sequences have a conserved, and highly shortened one-amino-acid intercysteine loop 4, whereas the chitin-binding 8C-HLPs have the SXΦXΦ motif at loop 3 and a six-amino-acid loop 4 with a conserved aromatic residue that is essential for chitin binding. Due to the absence of the chitin binding domain, ginsentides are non-chitin binding (Supplementary data S4).

The unique disulfide connectivity of ginsentides confers high stability against heat, proteolytic, acidic and human serum-mediated degradation. Chemical disulfide mapping and NMR analysis showed that three of four disulfide bonds of ginsentide TP1, Cys II-VI, III-VII, and V-VIII, are buried in the core of the structure. Consequently, the side chains of the other residues are all solvent-exposed, resulting in hydrophobic patches on the structural surface of the peptide. Thus, ginsentide TP1 displays an overall amphipathic distribution of the hydrophobic and hydrophilic side chains. The first residue in Cys I forms a disulfide bond with Cys IV, and the last residue in Cys VIII connects with Cys V. In this way, both the N- and C-termini of ginsentide TP1 are topologically fixed in the tertiary structure through disulfide bonds, which confers a pseudocyclic topology. This feature combined with a tightly folded structure fortified by four disulfide bonds and intramolecular hydrogen bonds, contribute to the high stability of ginsentides.

The biosynthetic precursors of ginsentides are also known as the mature product of ginseng-specific abundant proteins (GSAPs), which were previously identified in random gene screening of *Panax ginseng* and *Panax quinquefolius* genomes^40^. The mRNA transcripts of GSAPs were reported to be highly expressed in rhizomes, ranking third among 17,605 ESTs in the ginseng cDNA library. Biosynthesis of mature ginsentides from precursors is similar to that for other 8C-HLPs, which are generally synthesized as a three-domain precursor consisting of an N-terminal signal peptide, a mature peptide, and a C-terminal tail or a C-terminal protein-cargo. In this study, transcriptomic analysis showed that ginsentides are also biosynthesized as a three-domain precursor but have a different arrangement. The precursor architecture of ginsentides and ginsentide-like sequences consists of an N-terminal signal peptide, a pro-domain and a C-terminal mature peptide that differs from the protein-cargo family of chitin-binding 8C-HLPs. The processing of precursor proteins to mature ginsentides probably requires at least two proteolytic events. The first event is likely catalyzed by a signal peptidase that cleaves the ER signal peptide only after folding of the ginsentide domain by protein disulfide isomerases (PDIs) in the ER. The second event could be catalyzed by an unknown protease that targets a cleavage site at the N-terminal region of the ginsentide domain.

In conclusion, here we identified 14 novel ginsentides from *Panax ginseng*, *Panax quinquefolius*, and *Panax notoginseng* of the *Panax* family that have an unusual disulfide connectivity and represent a new precursor architecture that distinctly differs from all known 8C-HLPs. The novel and highly compact structure of ginsentides confers their resistance to heat, acid, and digestive enzymes. Ginsentides possess certain features of small chemical metabolites but have large footprints, which could be of interest for drug development. This study greatly expands the occurrence, disulfide connectivity, and precursor architectures of non-chitin binding 8C-HLPs.

## Materials and Methods

### Materials

All chemicals and solvents, unless otherwise stated, were purchased from Sigma Aldrich, US and Fisher Scientific, US.

### Isolation and purification of ginsentides

Dried roots, seeds, and flowers from *Panax ginseng*, *P*. *quinquefolius*, or *P*. *notoginseng* (Yue Hwa Chinese Products Emporium Ltd., Singapore) were pulverized and 100 mg were extracted with 0.5 mL 50% ethanol to screen for CRPs with molecular masses of 2–6 kDa by mass spectrometry using an Applied Biosystems 4800 MALDI TOF/TOF Analyzer. To obtain sufficient ginsentides for characterization studies, ~2 kg of dried material were extracted with 10 L water. The extracts were filtered and subjected to flash chromatography using C18 powder (Grace Davison). The ginsentide-enriched fractions were subsequently eluted with 60% ethanol and concentrated using a rotary evaporator. The concentrated fractions were then purified by preparative RP-HPLC using a C18 Grace Vydac column (250 × 22 mm) at a flow rate of 8 mL/min on a Shimadzu system. A linear gradient of 1%/min of 10–80% buffer B was applied. Buffer A contained 0.05% (v/v) trifluoroacetic acid (TFA) in HPLC grade water, and buffer B contained 0.05% (v/v) TFA and 99.5% (v/v) acetonitrile (ACN). To obtain isolated ginsentides, the resulting fractions were further purified by a semi-preparative C18 Vydac column (250 × 10 mm), using the same gradient, at a flow rate of 3 mL/min.

### Sequence determination

20 µg of isolated and purified ginsentides were dissolved in 50 µL 100 mM ammonium bicarbonate buffer (pH 7.8) containing 50% ethanol. *S*-reduction was performed with addition of 20 mM dithiothreitol (DTT) and incubated for 2 h at 37 °C. *S*-reduced ginsentides were *S*-alkylated with N-ethylmaleimide (NEM) followed by enzymatic digestion with trypsin or chymotrypsin at 37 °C. Peptide fragments were subjected to mass spectrometry and sequenced by MS/MS (Applied Biosystems 4800 MALDI TOF/TOF Analyzer) using nitrogen as the collision gas with an applied collision energy of 1 keV. Assignments of isobaric residues Ile/Leu and Lys/Gln of ginsentides were based on the nucleotide sequences obtained from the NCBI database.

### NCBI and Onekp Database search for ginsentide-like precursor sequences

TBLASTN and BLASTP was used to search for ginsentide-like precursor sequences in the NCBI and OneKp databases using the full precursor and mature ginsentide TP1-TP14 as query sequences with an expected value threshold of 100.

### Connectivity mapping

0.5 mg of Ginsentide TP1 was partially reduced in 2 mL 100 mM citrate buffer (pH 3.0) containing 20% ACN and 20 mM tris(2-carboxyethyl)phosphine (TCEP) at 37 °C for 40 min. Trapping of intermediates was done by adding excess NEM to a final concentration of 50 mM and incubating at 37 °C for 20 min. The reaction was quenched by immediate injection of samples into a C18 Vydac column (250 × 4.6 mm) at a flow rate of 1 mL/min. Intermediate species separated by RP-HPLC were analyzed by mass spectrometry to verify the number of NEM-alkylated cysteines. *S*-NEM intermediate species with one (1*SS*), two (2*SS*) or three (3*SS*) intact disulfide bonds were subsequently fully *S*-reduced with 20 mM DTT, and *S*-alkylated with 40 mM iodoacetamide (IAM). Mixed *S*-alkylated peptides were digested with trypsin and the resulting fragments were analyzed by MS/MS.

### NMR spectroscopy

Samples for NMR analysis were prepared by dissolving lyophilized ginsentide TP1 in 95% H_2_O/5% D_2_O or D_2_O directly (~1 mM protein and pH/pD 3.2). All NMR experiments were carried out on a Bruker 800 MHz NMR spectrometer equipped with a cryogenic probe. Two dimensional (2D) total correlation spectroscopy (TOCSY) and nuclear Overhauser spectroscopy (NOESY) experiments were performed with mixing times of 80 ms and 200 ms, respectively (49), to acquire two 2D data sets at 298 K and 303 K, respectively. Water suppression was achieved using modified WATERGATE pulse sequences (50). The NMR spectra were processed with NMRPipe software (51). The amides involved in hydrogen bonding were identified in the hydrogen-deuterium exchange one-dimensional (1D) ^1^H experiment (52).

### Resonance assignment

Sequence specific assignments were achieved based on the 2D TOCSY and NOESY, and NOEs were assigned from the 2D NOESY, using the in-house software NMRspy (http://yangdw.science.nus.edu.sg/Software&Scripts/NMRspy/index.htm). The chemical shifts were deposited in BioMagResBank under accession number 18983. Distance restraints were derived from the peak intensities of the assigned NOEs. Dihedral angles φ were obtained from 3JHN-Hα coupling constants measured from the 1D ^1^H spectrum. Hydrogen bond restraints were incorporated based on the observation of amide protons in the ^1^D ^1^H spectra recorded after re-suspending the lyophilized ginsentide TP1 in D_2_O for up to 18 h at 25 °C.

### Structure calculation

The solution structure was calculated using a simulated annealing approach with CYANA 2.0 (53). Distance restraints were divided into three classes: 1.8 < d ≤ 3.4 Å (strong NOEs), 1.8 < d ≤ 4.2 Å (medium NOEs) and 1.8 < d ≤ 5.5 Å (weak NOEs). Disulfide bond restraints of 2.0 ≤ d (Sγi, Sγj) ≤ 2.1 Å, 3.0 ≤ d (Cβi, Sγj) ≤ 3.1 Å, and 3.0 ≤ d (Sγi, Cβj) ≤ 3.1 Å were used for structure calculation. During the structure calculation, hydrogen bond restraints of 1.8–2.2 Å for the NH-O distance, and 2.2 to 3.2 Å for the HN-O distance were applied on nine identified hydrogen bonds according to the slowly exchanging amide protons. Φ angles were constrained to the range of −150° to −90° for 3JHN-Hα >8 Hz. Structures were displayed and analyzed using software Pymol (http://www.pymol.org) and program PROCHECK-NMR, respectively (54). The structure was deposited with a PDB code: 2ML7.

### Chitin binding assay

Ginsentide TP1 was incubated with chitin beads (New England Biolabs, Ipswich, MA US) in chitin binding buffer (10 mM phosphate; pH 7.4) at room temperature for 1 h. At each time point up to 1 h, the beads were centrifuged at 12,000 g for 1 min and the absorbance of the supernatant was read at 214 nm to assess binding. Samples were further analyzed by MALDI-TOF MS.

### Stability assays

#### Heat Stability

10 μg ginsentide TP1 was dissolved in 100 μL distilled water and incubated at 100 °C for 30, 60, 90, and 120 min. As a control, a replica was performed with incubation at room temperature. The RP-HPLC profiles of the heated and control samples were compared to evaluate their stability.

#### Enzymatic Stability

10 μg ginsentide TP1 was dissolved in 100 μL 100 mM ammonium bicarbonate buffer (pH 7.8) with 1 μL 0.5 μg/μL trypsin or chymotrypsin, incubated at 37 °C for 3 h. Stability assays against pepsin was performed with ginsentide TP1 dissolved in 100 mM sodium citrate buffer (pH 2.5). A replica without enzymes served as the control. The RP-HPLC profiles of the treated and control samples were compared to evaluate their stability.

#### Acid Stability

10 μg ginsentide TP1 was dissolved in 100 μL 0.2 M HCl and incubated at 37 °C for 2 h. A control replica was performed without the addition of acid. The RP-HPLC profiles of the treated and control samples were compared to evaluate their stability.

#### Human serum-mediated stability

0.1 mM ginsentide TP1 was incubated in 25% human serum in Dulbecco’s Modified Eagle Medium (DMEM) (GE Healthcare Life Sciences, UK) containing 1 mM sodium pyruvate, 4 mM L-glutamine, without phenol red at 37 °C for 48 h. Synthetic peptide DALK (sequence: KRPPGFSPL) was used as a positive control. After incubation, precipitation was performed with an addition of 100% ethanol and centrifuged at 18,000 g for 15 min, 4 °C. Supernatants were collected in a fresh tube and monitored using analytical RP-HPLC (Shimadzu Shim-pack XR-C8 column, 3.0 × 50 mm, 2.2 µm, flow rate 0.3 mL/min, Japan), with a 30 min linear gradient of 0–50% buffer B (0.05% TFA (v/v) in 99.5% ACN). Individual peaks were collected and identified by MALDI-TOF MS.

### Cell culture

Huh7 (human liver carcinoma cells) and human-derived endothelial cells (HUVEC-CS) were kindly provided by Professor Kathy Qian Luo (Nanyang Technological University, Singapore). THP-1 cells were cultured in DMEM or RPMI medium (Thermo Scientific HyClone) supplemented with 10% fetal bovine serum, 100 U/mL of penicillin and streptomycin and grown in a 5% CO_2_ humidified incubator at 37 °C.

### Cell viability assay

Cell viability was measured using a 3-(4,5-dimethylthiazolyl-2)−2,5-diphenyltetrazolium bromide (MTT) dye reduction assay. Briefly, cells were treated with ginsentide TP1 or 0.1% Triton X-100 (positive control) for 24 h. MTT (final concentration 0.5 mg/mL) was added and incubated for 3 h at 37 °C. Dimethyl sulfoxide was then added to dissolve insoluble formazan crystals. The absorbance was measured at 550 nm using a microplate reader (Tecan Infinite® 200 Pro, Switzerland).

### Hemolytic assay

Red blood cells were washed three times and re-suspended in PBS to give a final 1% suspension. 95 µL of the 1% suspension was added to each well of a 96-well plate. Ginsentide TP1 was serially diluted in PBS, and 5 µL of the peptide samples was added into the wells at final concentrations of 6.25, 12.5, 25, and 50 μM. Each concentration was tested in triplicate. The plate was incubated at 37 °C for 1 h and centrifuged at 1,000 rpm for 6 min. 60 µL of the supernatant were transferred to a new 96-well plate. The absorbance was measured at 415 nm. The level of hemolysis was calculated as the percentage of maximum lysis (1% Triton X-100 control) after adjusting for minimum lysis (PBS control).

### Immunogenicity assay

Approximately 1 × 10^6^ THP-1 cells were seeded into each well of 12-well plates. The cells were treated with ginsentide TP1 for 6 h with lipopolysaccharide as the positive control. The supernatants were collected and stored at −80 °C until measurement. The concentrations of TNF-α, IL-6, IL-8 and IL-10 were determined by enzyme-linked immunosorbent assay using ELISA MAX™ Deluxe Sets (BioLegend, USA).

### Statistical analyses

Statistical comparisons were performed using GraphPad Version 6.0d (USA). The data were analyzed by one way analysis of variance (ANOVA) followed by Newman-Keuls post hoc test. Data were expressed as mean ± S.E.M. P < 0.05 was considered statistically significant.

## Electronic supplementary material

Supplementary Data Set 1
