# Supplementary material for: Ginsentides: Cysteine and Glycine-rich Peptides from the Ginseng Family with Unusual Disulfide Connectivity
Source: Sci Rep. 2018 Nov 1;8:16201. doi: 10.1038/s41598-018-33894-x (PMC6212409; doi:10.1038/s41598-018-33894-x)
Supplement: Supplementary file 1 — Supplementary Data Set 1 [file 41598_2018_33894_MOESM1_ESM.docx]

**Ginsentides: Cysteine and Glycine-rich Peptides from the Ginseng Family with Unusual Disulfide Connectivity**

**James P. Tam ^1*^, Giang K. T. Nguyen^1^, Shining Loo^1^, Shujing Wang^1^, Daiwen Yang^2^, and Antony Kam^1^**

^1^ School of Biological Sciences, Nanyang Technological University, 60 Nanyang Drive, 637551, Singapore
^2^ Department of Biological Sciences, National University of Singapore, 14 Science Drive 4, 117543, Singapore

​

________________________

^*^Corresponding author: Professor James P. Tam, School of Biological Sciences, Nanyang Technological University, 60 Nanyang Drive, 637551, Singapore

Email: JPTam@ntu.edu.sg

**
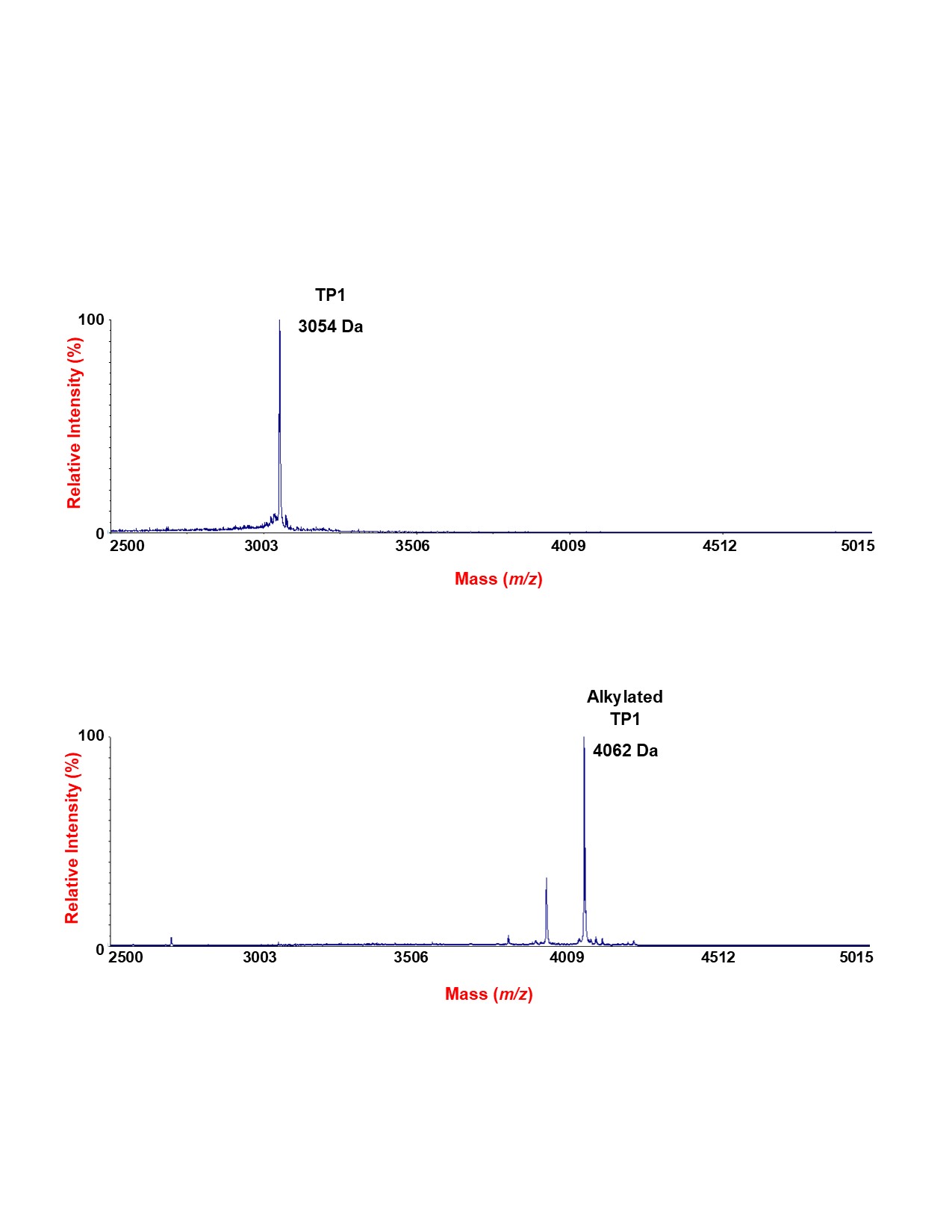
**

**Supplementary Data S1.** Mass spectrometry profile of ginsentide TP1 before and after *S*-reduction and *S*-alkylation.


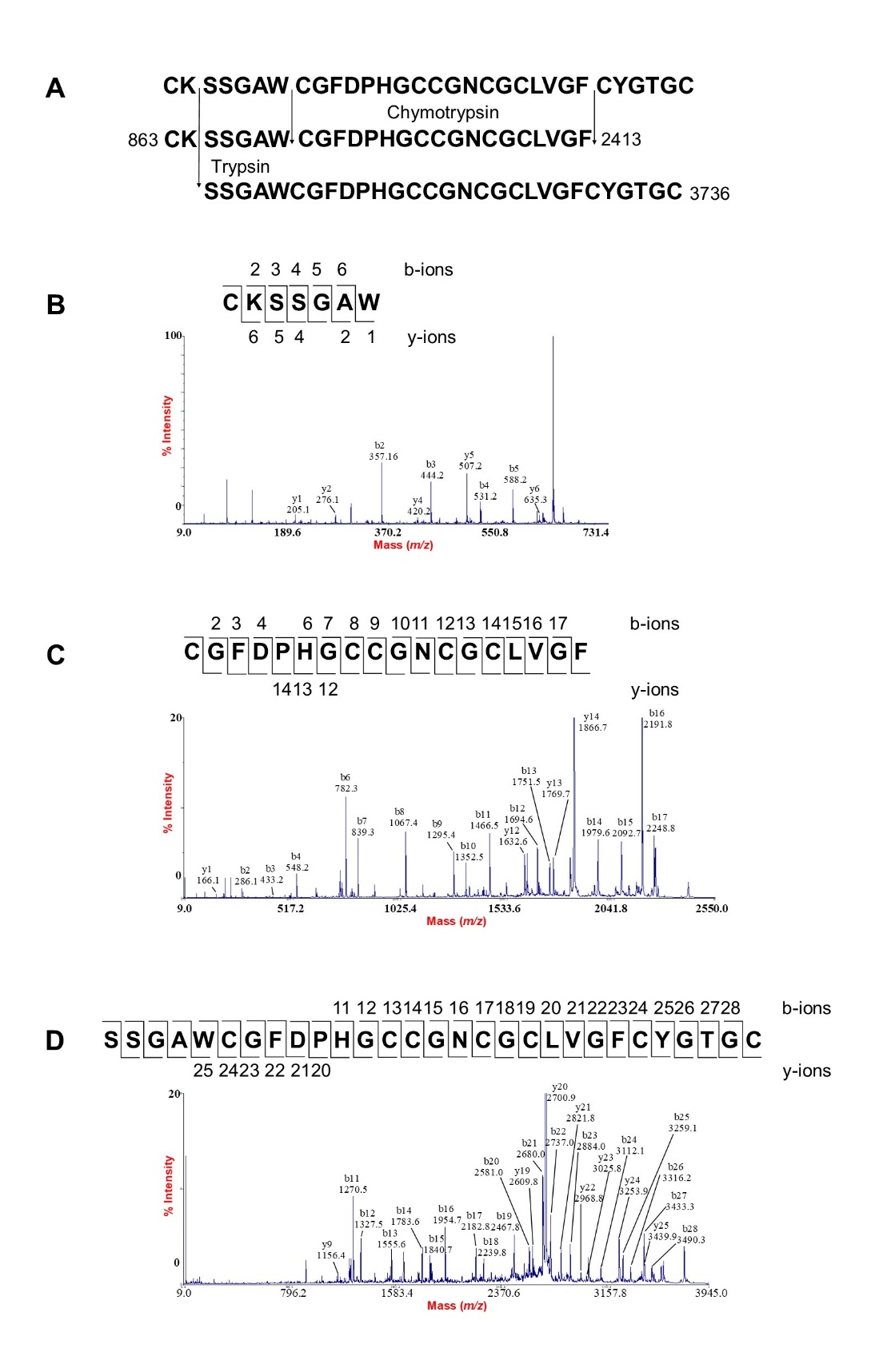


**Supplementary Data S2. *De novo* sequencing of ginsentide TP2.** **(A)** Enzymatic digestion of S-alkylated peptides by chymotrypsin and trypsin generated three fragments with m/z values of 863, 2413, and 3736, respectively. The sequence of fragments were deduced using the b-ions and y-ions generated from MALDI-TOF MS/MS. **(B)** MS/MS spectra of the 863 Da fragment; **(C)** MS/MS spectra of the 2413 Da fragment; **(D)** MS/MS spectra of the 3736 Da fragment.


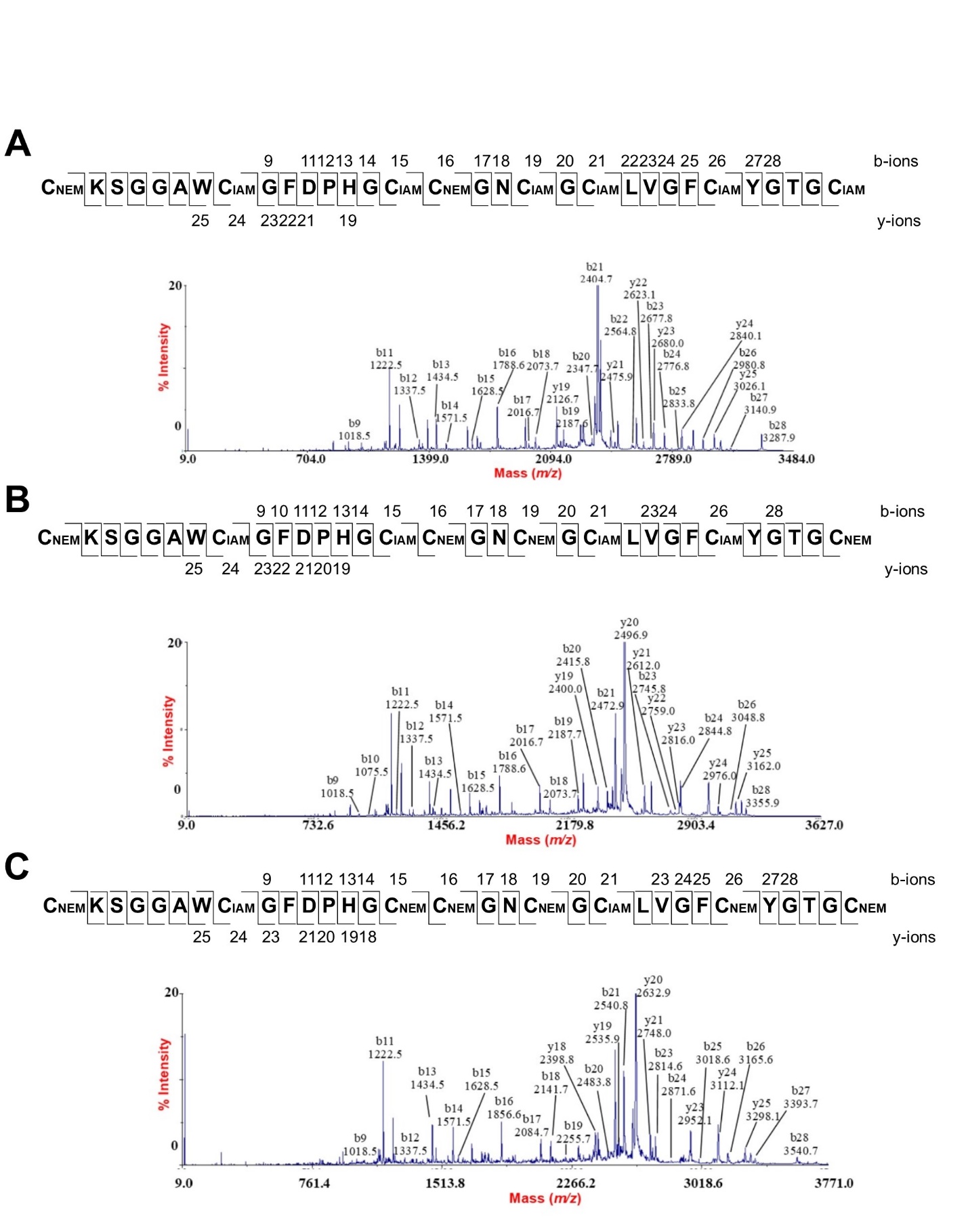


**Supplementary Data S3. *De novo* sequencing of the partially *S*-reduced and *S*-alkylated ginsentide TP1 (A)** 3*SS,* **(B)** 2*SS*, and **(C)** 1*SS* species.

**
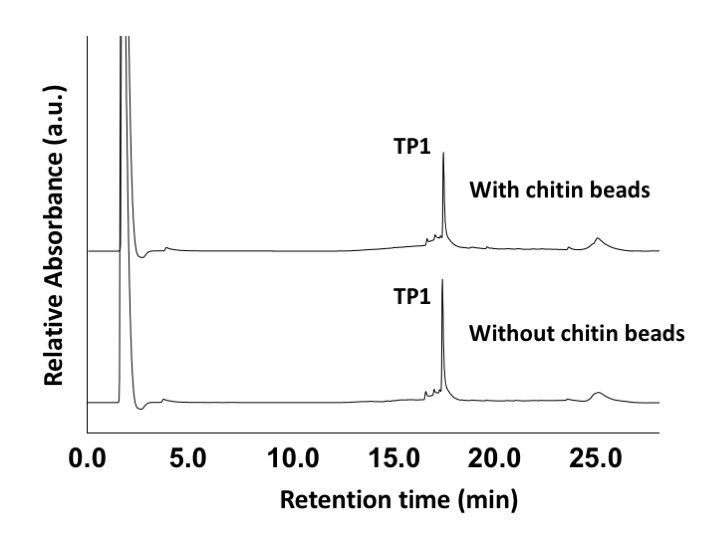
Supplementary Data S4. HPLC profile of the chitin-binding properties of ginsentide TP1.**
